# Mechanical stretch promotes apoptosis and impedes ciliogenesis of primary human airway basal stem cells

**DOI:** 10.1186/s12931-023-02528-w

**Published:** 2023-09-29

**Authors:** Li-Qin Lin, Hai-Kang Zeng, Yu-Long Luo, Di-Fei Chen, Xiao-Qian Ma, Huan-Jie Chen, Xin-Yu Song, Hong-Kai Wu, Shi-Yue Li

**Affiliations:** 1 The First Affiliated Hospital of Guangzhou Medical University, Guangzhou, 510120 Guangdong China; 2 National Clinical Research Center for Respiratory Disease, Guangzhou, 510120 Guangdong China; 3 Guangzhou Institute of Respiratory Health, Guangzhou, 510120 Guangdong China; 4 State Key Laboratory of Respiratory Disease, Guangzhou, 511495 Guangdong China; 5 The Fifth Affiliated Hospital of Guangzhou Medical University, Guangzhou, 510799 Guangdong China; 6 Key Laboratory of Biological Targeting Diagnosis, Guangzhou, 510799 Guangdong China; 7 Therapy and Rehabilitation of Guangdong Higher Education Institutes, Guangzhou, 510799 Guangdong China; 8 Innovation Centre for Advanced Interdisciplinary Medicine, Guangzhou, 510799 Guangdong China

**Keywords:** Airway basal stem cells, Mechanical stretch, Ciliogenesis

## Abstract

**Background:**

Airway basal stem cells (ABSCs) have self-renewal and differentiation abilities. Although an abnormal mechanical environment related to chronic airway disease (CAD) can cause ABSC dysfunction, it remains unclear how mechanical stretch regulates the behavior and structure of ABSCs. Here, we explored the effect of mechanical stretch on primary human ABSCs.

**Methods:**

Primary human ABSCs were isolated from healthy volunteers. A Flexcell FX-5000 Tension system was used to mimic the pathological airway mechanical stretch conditions of patients with CAD. ABSCs were stretched for 12, 24, or 48 h with 20% elongation. We first performed bulk RNA sequencing to identify the most predominantly changed genes and pathways. Next, apoptosis of stretched ABSCs was detected with Annexin V-FITC/PI staining and a caspase 3 activity assay. Proliferation of stretched ABSCs was assessed by measuring *MKI67* mRNA expression and cell cycle dynamics. Immunofluorescence and hematoxylin-eosin staining were used to demonstrate the differentiation state of ABSCs at the air-liquid interface.

**Results:**

Compared with unstretched control cells, apoptosis and caspase 3 activation of ABSCs stretched for 48 h were significantly increased (p < 0.0001; p < 0.0001, respectively), and *MKI67* mRNA levels were decreased (p < 0.0001). In addition, a significant increase in the G0/G1 population (20.2%, p < 0.001) and a significant decrease in S-phase cells (21.1%, p < 0.0001) were observed. The ratio of Krt5^+^ ABSCs was significantly higher (32.38% vs. 48.71%, p = 0.0037) following stretching, while the ratio of Ac-tub^+^ cells was significantly lower (37.64% vs. 21.29%, p < 0.001). Moreover, compared with the control, the expression of *NKX2-1* was upregulated significantly after stretching (14.06% vs. 39.51%, p < 0.0001). RNA sequencing showed 285 differentially expressed genes, among which 140 were upregulated and 145 were downregulated, revealing that *DDIAS, BIRC5, TGFBI*, and *NKX2-1* may be involved in the function of primary human ABSCs during mechanical stretch. There was no apparent difference between stretching ABSCs for 24 and 48 h compared with the control.

**Conclusions:**

Pathological stretching induces apoptosis of ABSCs, inhibits their proliferation, and disrupts cilia cell differentiation. These features may be related to abnormal regeneration and repair observed after airway epithelium injury in patients with CAD.

**Supplementary Information:**

The online version contains supplementary material available at 10.1186/s12931-023-02528-w.

## Background

Airway epithelium functions as the first line of respiratory tract defense against various environmental insults. Airway epithelial damage usually occurs in chronic airway diseases (CAD), such as asthma, chronic obstructive pulmonary disease (COPD), and bronchiectasis. Maintaining pulmonary homeostasis is crucial to restore the structural integrity and function of the airway epithelium. Airway basal stem cells (ABSCs), located adjacent to airway epithelial basal lamina, are a population of cells with self-renewal and differentiation abilities. ABSCs are capable of generating ciliated cells, secretory cells, and neuroendocrine cells [1–4]. Accordingly, serving as the resident stem cells of the airway, ABSCs dominate processes to maintain the dynamic balance of normal airway epithelium and repair after injury [4]. Various insults or repeated injuries could cause hyperplasia of ABSCs or dysplasia of airway epithelial cells, both of which are associated with the occurrence and development of CAD [5, 6]. Rao et al. found that abnormal ABSCs in COPD resulted in pathologic characteristics including airway fibrosis, inflammation, and mucus hypersecretion [7]. Peng et al. reported that airway progenitor cells were abnormally proliferative in bronchiectasis [8]. Moreover, growing evidence indicates that the dysfunction of ABSCs plays a crucial role in driving the progression of idiopathic pulmonary fibrosis [9]. However, the mechanism and function of ABSCs underlying the development of chronic inflammatory diseases remain unclear.

Mechanical stretch plays a pivotal role in lung development and disease progression. Respiratory diseases can alter the mechanical environment of the airway to regulate its structure and function [10–12]. In patients with CAD (e.g., COPD, bronchiectasis, or pulmonary fibrosis resulting from various factors), bronchial/alveolar structure destruction, mucus hypersecretion, and constriction of airway smooth muscle led to mechanical stress in the airway continuously and abnormally elevated; moreover, these individuals display ABSCs with dysfunctional self-renewal and regeneration [10, 13]. Further, the high-level mechanical stress exerted on the airway epithelium involved critical pathological processes, including inducing the expression of inflammatory mediators, such as interleukin (IL)-8, IL-13, and matrix metalloproteinase 9 (MMP-9), inhibiting epithelial repair and even causing the airway remodeling [14–20]. These researches suggested that mechanical stress probably influenced the normal physiological function of ABSCs [17, 21, 22]. Therefore, we used a Flexcell® FX-5000 Tension System (Flexcell International Corporation, Hillsborough, NC, USA), which can mimic the pathological airway mechanical stretch conditions of patients with CAD, to examine the effect of mechanical stretch on primary human ABSCs’ function. Finally, we explored the role of mechanical stretch in the repair and regeneration abilities of ABSCs.

## Methods

### ABSCs culture

Airway epithelium samples were collected by brushing the contralateral grade 3–5 bronchus of patients with solitary pulmonary nodules using a flexible fiberoptic bronchoscope (Olympus Corporation, Tokyo, Japan), the information of patients is provided in Additional file 1 (see Table S2). ABSCs were isolated and cultured from airway epithelium samples according to a previously published method [23]. The identification of ABSCs see also Figure S1. The medium was changed every 2 or 3 days. Cells were passaged at 80% confluency and used between the third to sixth passages in the following experiments. The study was approved by the ethics committee of the First Affiliated Hospital of Guangzhou Medical University (Ethics Review Number 2022 − 138). Informed consent was obtained from all study participants.

### Mechanical stretch

ABSCs were inoculated onto collagen I-coated Bioflex® six-well culture plates at a density of 2 × 10^5^ cells per well and cultured for 24 h to reach 60–70% confluence. ABSCs were then divided into control (unstretched) and stretched groups. The experimental group was exposed to mechanical stretch stimulation (loading parameters: 0.33 Hz frequency, sine wave, 20% elongation) for 12, 24, and 48 h using a Flexcell FX-5000 Tension System to simulate pathological conditions related to mechanical stress in the airway [18, 21, 24]. The control group was placed in the same 37ºC, 5% CO_2_ incubator without any additional intervention. Three independent wells/group were performed.

### Apoptosis assay

#### Cell apoptosis

Cell apoptosis was measured using an Annexin V-FITC/PI apoptosis kit (556,547; BD Pharmingen, NJ, USA). Harvested ABSCs were stained with 100 µL of 1× binding buffer following incubation with 5 µL of FITC-Annexin V and 5 µL of propidium iodide (PI) for 15 min at room temperature in the dark. Ratios of apoptotic cells were detected using an LSRFortessa X-20 Flow Cytometer (Becton-Dickinson Immunocytometry Systems, San Jose, CA, USA).

#### Caspase-3 activity assay

ABSCs were harvested and washed with PBS. Next, 50 µL of lysis buffer was added to lyse cells for 15 min on an ice bath. The supernatant released from these lysed cells was collected. According to the manufacturer’s instructions, caspase-3 activity was detected using a detection kit (Beyotime Institute of Biotechnology, Shanghai, China). This kit is based on the ability of caspase-3 to catalyze acetyl-Asp-Glu-Val-Asp p-nitroanilide to produce yellow p-nitroaniline, which has a strong absorption at 405 nm. Thus, the activity of caspase-3 is reflected by measuring the absorbance value (optical density value).

### Cell cycle

ABSCs were fixed with ice-cold 70% ethanol (4ºC overnight). Fixed ABSCs were washed once with cold PBS and stained in 450 µL of PI (KGA512; KeyGEN Biotech, China). Next, 50 µL of RNase A was added and incubated for 30 min at room temperature in the dark. Samples were immediately analyzed on an LSRFortessa X-20 Flow Cytometer, with 5 × 10^4^ events acquired at 488 nm. Finally, percentages of cells in G0/G1, S, and G2/M phases of the cell cycle were analyzed using FlowJo software 10.8.1 (Treestar, Ashland, OR, USA) on a MAC1 workstation.

### RNA extraction and quantitative real-time PCR (RT‑qPCR)

TransZol Up Plus RNA Kit (ER501-01; TransGen Biotech, Beijing, China) was used to extract total RNA from control and stretched ABSCs. cDNA was synthesized from all RNA samples using Hifair® III 1st Strand cDNA Synthesis SuperMix for qPCR (gDNA Digester Plus) reverse transcription reagents (11141ES60; Yeasen, Shanghai, China) according to the manufacturer’s protocol. Expression of target gene mRNA in human ABSCs was detected by RT‑qPCR. RT‑qPCR detection was performed using a CFX ConnectTM Real-Time PCR Detection System (Bio-Rad, Hercules, CA, USA) with Hieff UNICON® Power qPCR SYBR Green Master Mix (11195ES08*, Yeasen). RT-PCR conditions were as follows: 40 cycles of predenaturation at 95 °C for 30 s, denaturation at 95 °C for 10 s, annealing at 60 °C for 20 s, and extension at 72 °C for 20 s. *GAPDH* was used as the endogenous control gene and expression levels of target genes were quantified using the comparative threshold cycle method with arithmetic formulae. Primer sequences for RT‑qPCR are provided in the Additional files (see Table S1).

### Air-Liquid Interface (ALI) Cultures

ABSCs were seeded onto a Transwell insert (3470, Corning) at a density of 1–2 × 10^5^ cells/well in 200 µL of ALI media, which was a 1:1 mixture of DMEM and BEGM (CC-3170; Lonza, Walkersville, USA). When cell confluence was reached, the apical medium was removed and 800 µL of ALI medium was added to the basal chamber only and changed every 2 days. After 21 days of differentiation, ALI cultures were fixed with 4% paraformaldehyde at room temperature for 15 min and then embedded in paraffin. Three independent wells/group were measured.

### Immunofluorescence and hematoxylin and eosin staining

Paraffin sections were deparaffinized by placing them 2 h on a 60–70ºC heating block, followed by a series of Histo-Clear and ethanol washes. Antigen retrieval was performed with 10 mM sodium citrate buffer (pH6) using a pressure cooker for 10 min. Sections were blocked in 5% bovine serum albumin (130-091-376-1; Miltenyi Biotec, Bergisch Gladbach, Germany) for 30 min at room temperature, followed by incubation with primary antibody at 4ºC overnight. Secondary antibodies were diluted in phosphate buffer and added to the sections to incubate for 1 h in the dark. Next, sections were counterstained with DAPI (G1012-10ML; Servicebio, Wuhan, China). The negative control was stained with secondary antibodies only. Images were captured and analyzed using an inverted fluorescence microscope (DMi8; Leica Wetzlar, Germany). Primary antibodies included anti-acetylated tubulin (1:500; T6793; Sigma-Aldrich, St. Louis, MO, USA), anti-Cytokeratin 5 (1:200, ab52635-100, Abcam, Cambridge, UK), anti-NKX2-1 (1:25, ab227652, Abcam, Cambridge, UK), goat Anti-Rabbit IgG (1:200, ab150080, Abcam, Cambridge, UK), goat anti-mouse IgG (1:200; ab150115-500ug; Abcam, Cambridge, UK), and DAPI. Paraffin sections were stained with Hematoxylin and eosin according to the instructions.

### RNA sequencing (RNA-Seq) analysis (transcriptome profiling)

We harvested 2 × 10^6^ ABSCs from unstretched and 48 h stretched groups to perform RNA-Seq. Every group contained three biological replicates. According to the manufacturer’s protocol, total RNA was extracted using a Trizol reagent kit (Invitrogen, Carlsbad, CA, USA). RNA quality was assessed on an Agilent 2100 Bioanalyzer (Agilent Technologies, Palo Alto, CA, USA) and checked using RNase-free agarose gel electrophoresis. After total RNA was extracted, eukaryotic mRNA was enriched by Oligo(dT) beads, while prokaryotic mRNA was enriched by removing rRNA with a Ribo-Zero™ Magnetic Kit (Epicentre, Madison, WI, USA). Next, the enriched mRNA was fragmented into short fragments using a fragmentation buffer and reverse transcribed into cDNA with random primers. Second-strand cDNA was synthesized by DNA polymerase I, RNase H, dNTP, and buffer. Next, the cDNA fragments were purified with a QiaQuick PCR Extraction Kit (Qiagen, Venlo, the Netherlands), end-repaired, poly(A) added, and ligated to Illumina sequencing adapters (Illumina, San Diego, CA, USA). Ligation products were size-selected by agarose gel electrophoresis, PCR-amplified, and sequenced using Illumina HiSeq2500 by Gene Denovo Biotechnology (Guangzhou, China).

### Protein - protein interaction (PPI) analysis

We first picked the differentially expressed genes (DEGs) involved in the biological processes of epithelial cell differentiation and regulation of cell differentiation in Gene Ontology analysis. The relationship of these genes, such as co-expression and co-localization, and the PPI network were obtained based on STRING software (https://string-db.org/).

### Statistical analysis

All statistical analyses were performed using GraphPad Prism 8 (GraphPad Software, San Diego, CA, USA). Data are presented as the mean ± standard deviation. ANOVA or unpaired t-test was used to analyze differences between experimental groups. An alpha value of < 0.05 was considered statistically significant.

## Results

### Developmental timing of mechanical stretch-inducible apoptosis of ABSCs

**Fig. 1:**
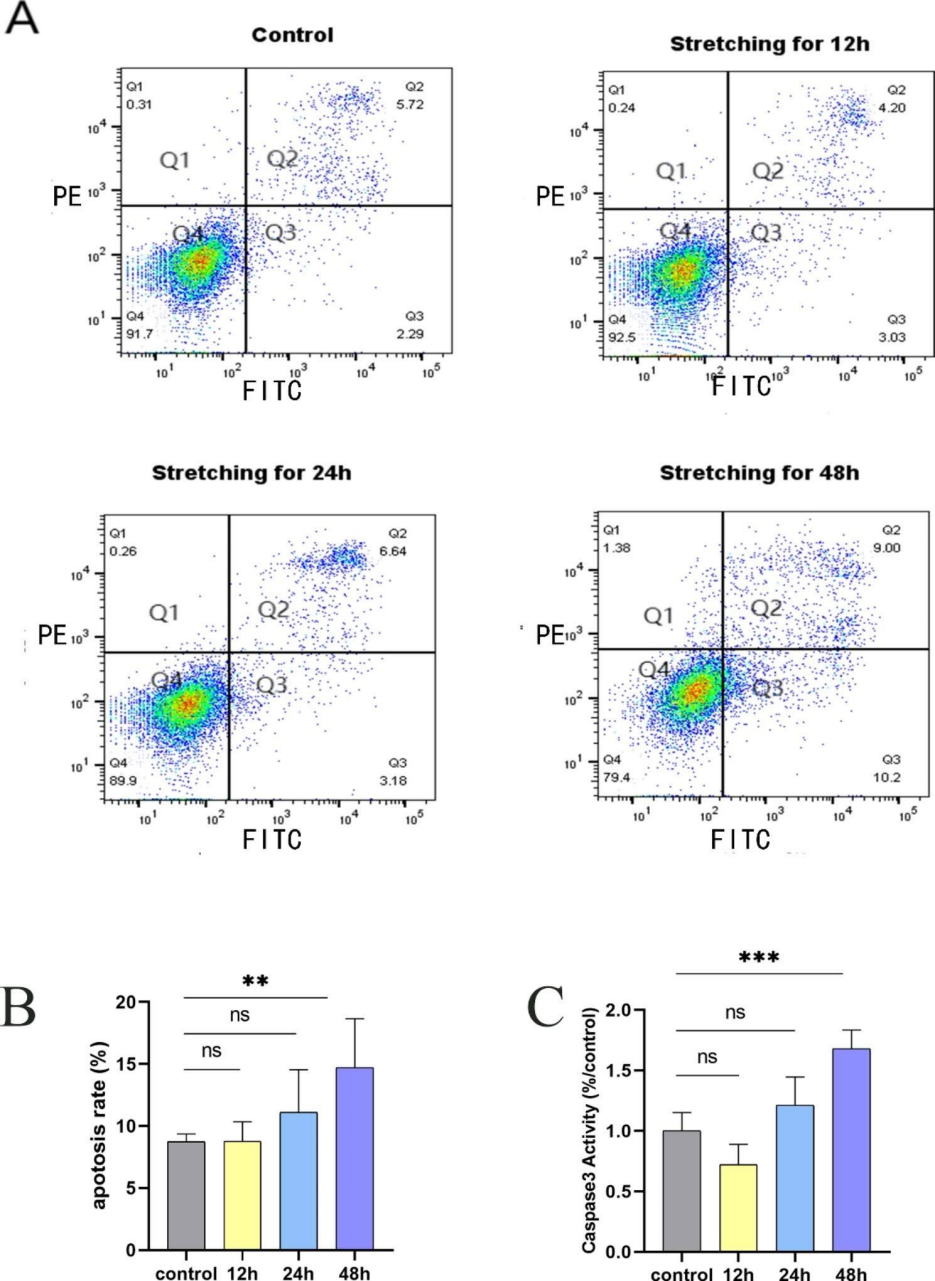
Mechanical stretching for 48 h induced the apoptosis ABSCs. (**a**) Cell apoptosis was detected by an annexin V FITC/ propidium iodide (PI) staining and flow cytometry analysis. Live cells (Q4) were stained negative for both annexin V and PI. Late apoptotic cells (Q2)were stained positive for both annexin V and PI. Early apoptotic cells (Q3) were stained positive for annexin V and negative for PI. Necrotic cells (Q1) were stained negative for annexin V and positive for PI. The apoptosis rate included Q2 and Q3. (**b**) Data are presented as the mean ± SD. **P < 0.01 compared with the control group. (**c**) Mechanical stretching for 48 h induced the activation of caspase 3 enzyme. Data are expressed as the mean ± SD from at least three experiments. ***P < 0.001 compared with the control group

We evaluated the effects of different durations of stretching on ABSCs apoptosis using Annexin V-FITC/PI staining. We found that with increased duration of stretching, apoptosis of ABSCs increased. When ABSCs were stretched for 48 h, a significant stretch-inducible increase in apoptosis was observed compared with the control (19.2% vs. 8.0%, p < 0.0001) (Fig. 1A-B). In addition, we used a colorimetric enzyme assay to detect the activity of caspase-3, a key enzyme in the process of apoptosis [25]. Similarly, compared with the control, levels of caspase-3 enzyme activity induced by stretch increased in a time-dependent manner, especially with stretching for 48 h (p < 0.0001) (Fig. 1C). However, no apparent difference could be seen in ABSCs stretched for 12 or 24 h compared with the control.

### Mechanical stretch inhibited ABSCs proliferation by inducing cell cycle arrest in G1å

**Fig. 2:**
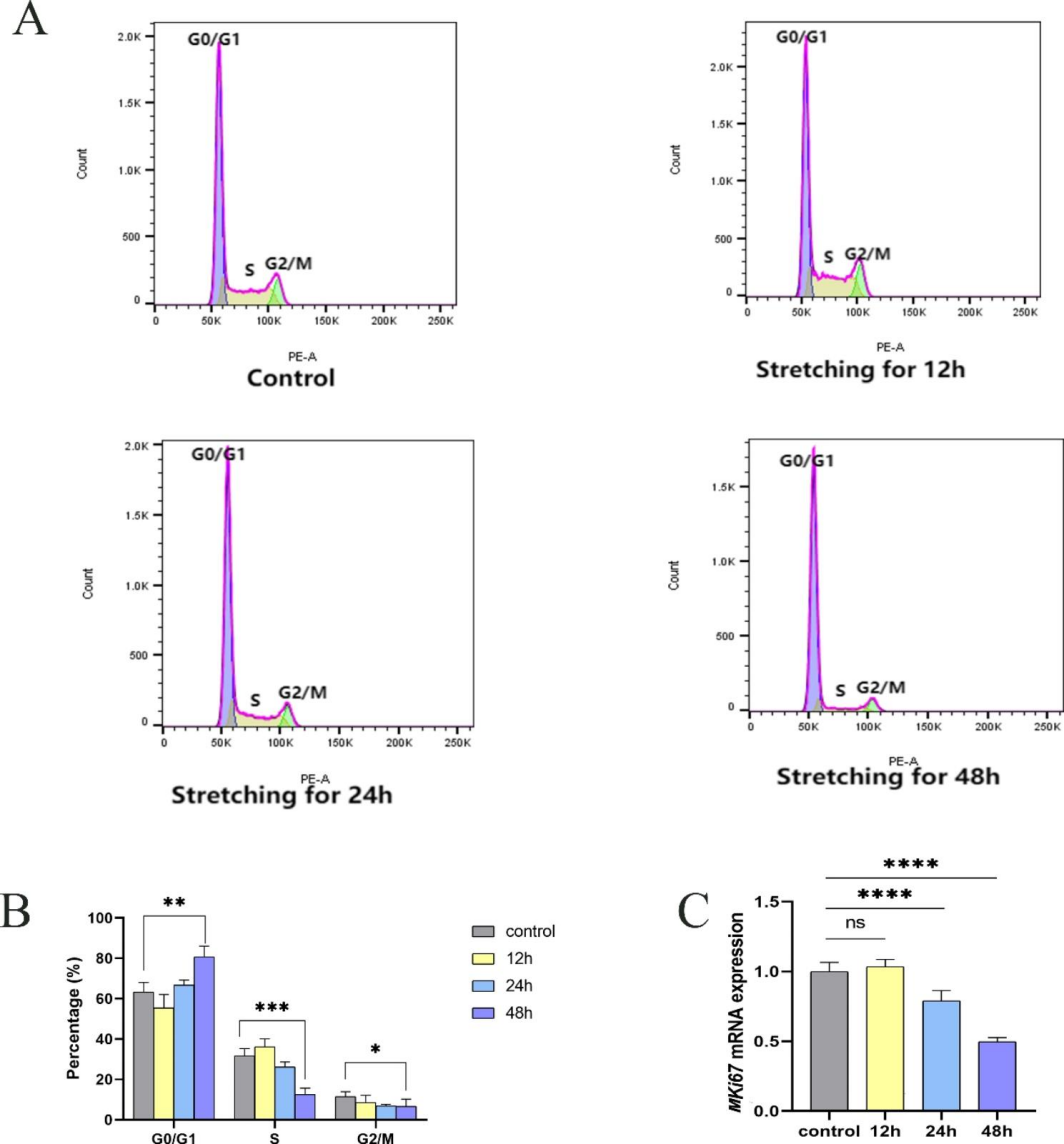
Mechanical stretching for 48 h induced the G1 arrest in ABSCs. (**a**) The percentage of cells in G0/G1, S, and G2/M phases of the cell cycle was analyzed by flow cytometry. (**b**) Control vs. stretch *P < 0.05, **P < 0.01, ***P < 0.001. Results are expressed as mean ± SD. (**c**) Mechanical stretching for 48 h decreased the mRNA expression of *MKI67* in ABSCs. Data are expressed as the mean ± SD from three different experiments. ****P < 0.0001 compared with the control group

The self-renewal ability of ABSCs is crucial for maintaining and restoring airway homeostasis. Therefore, we investigated the effects of mechanical stretch on ABSCs proliferation by evaluating expression levels of *MKI67* mRNA, a marker of proliferation. RT-qPCR data reveal significantly lower expression of *MKI67* mRNA in ABSCs stretched for 24 and 48 h (p < 0.0001, and p < 0.0001, respectively) compared with unstretched ABSCs, but there was no change for 12 h (Fig. 2C). We further studied the cell cycle dynamics of ABSCs proliferation under stretch. As the duration of stretching prolonged, the G0/G1 population of ABSCs increased gradually, while S-phase and G2/M populations decreased gradually. When ABSCs were stretched for 48 h, an apparent 20.2% increase in the G0/G1 population and remarkable 21.1% decrease in S-phase cells was observed compared with the control (p < 0.001 and p < 0.0001, respectively), indicating that a prolonged duration of mechanical stretch could induce G1 arrest and prevent cell cycle progression into S phase (Fig. 2A-B; Table 1). Similarly, there was no significant difference between ABSCs stretched for 12 and 24 h.

**Table 1:** The dynamic of cell cycle of ABSCs at different times induced by mechanical stretch

Group	G0/G1phase	S phase	G2/M phase
**Control**	63.3 ± 4.8	31.6 ± 3.7	11.5 ± 2.4
**Stretch**			
**12 h**	55.4 ± 6.8	36.1 ± 4.1	8.5 ± 3.6
**24 h**	66.8 ± 2.5	26.2 ± 2.4	6.8 ± 0.8
**48 h**	83.5 ± 0.8**	10.5 ± 0.3***	5.2 ± 1.4*

Data were presented as mean ± SD from at least three experiments. *p < 0.05, **p < 0.01, ***p < 0.001 compared with the control group

### Mechanical stretch impaired the ability of ABSCs to differentiate into ciliated cells

**Fig. 3:**
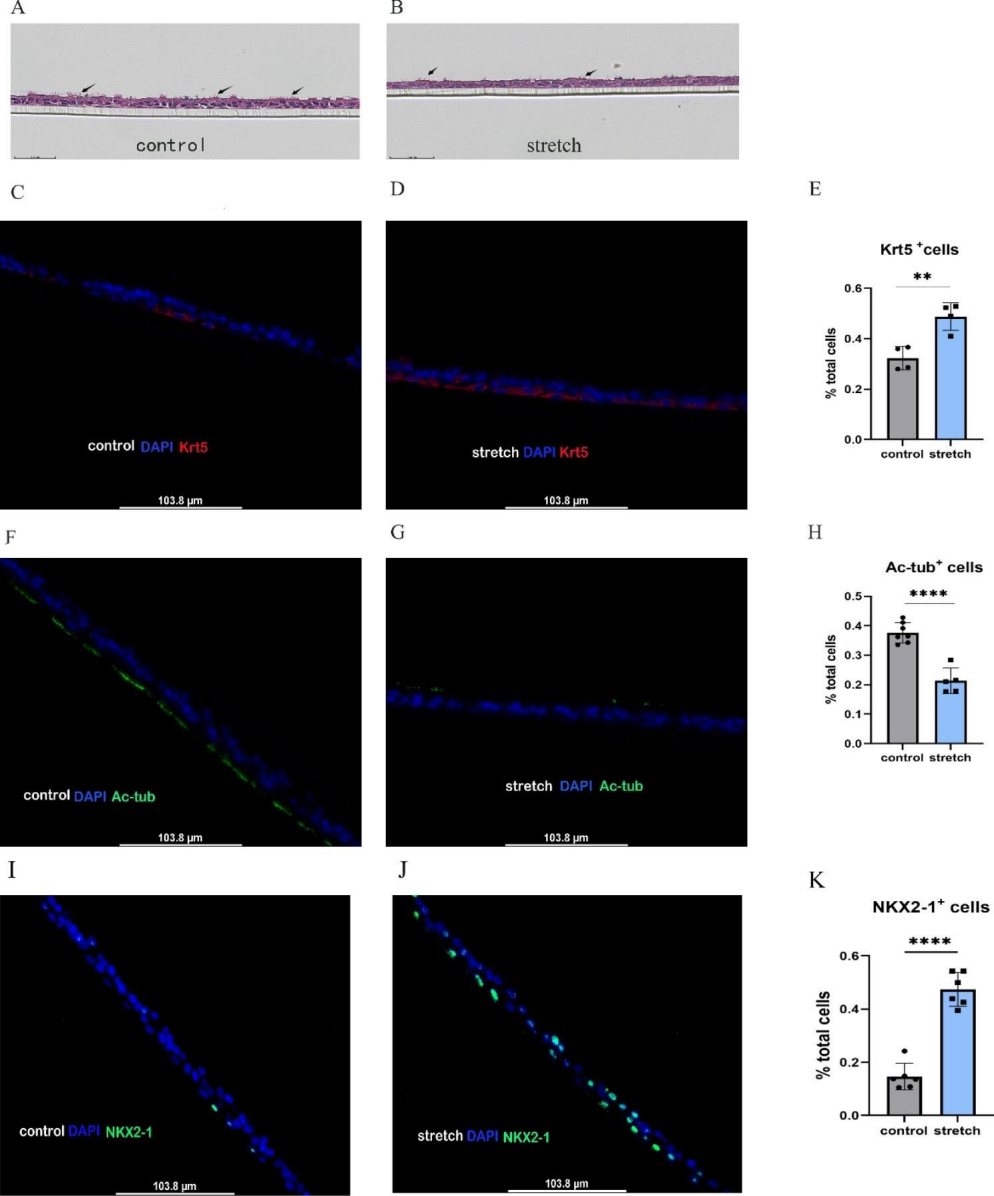
Mechanical stretch impaired the ability of ABSCs to differentiate into ciliated cells. (**a**) H&E staining of air-liquid interface (ALI) culture sections in control unstretched ABSCs, black arrow: ciliated cells. (**b**) H&E staining of ALI culture sections in stretched ABSCs, black arrow: ciliated cells. (**c**) Control unstretched ABSCs stained with anti-Krt5 (red) and DAPI (blue). (**d**) Stretched ABSCs stained with anti-Krt5 (red) and DAPI (blue). (**e**) Frequency of cells expressing Krt5 in the control and stretched groups. (**f**) Control unstretched ABSCs stained with anti-Ac-tub (green) and DAPI (blue). (**g**) Stretched ABSCs stained with anti-Ac-tub (green) and DAPI (blue). (**h**) Frequency of cells expressing Ac-tub in the control and stretched groups. (**I**) Unstretched ABSCs stained with anti-NKX2-1 (green) and DAPI (blue). (**J**) Stretched ABSCs stained with anti-NKX2-1 (green) and DAPI (blue). (**K**) Frequency of cells expressing NKX2-1 in the control and stretched groups. Representative fluorescence microscopy images from three separate experiments. Scale bar represents 103.8 μm in (c), (d), (f), (g), (I) and (J), Compare with the control, **P < 0.01, ****P < 0.0001

We further investigated the performance of ABSCs differentiation after stretching by air-liquid interface culture. Expression of differentiation markers was assessed with a fluorescence microscope. Our results demonstrate that after mechanical stretching of ABSCs for 48 h, remarkedly more differentiated cells were positive for the ABSCs marker keratin 5 (*Krt5*), compared with the control (48.71% vs. 32.38%, p = 0.0037) (Fig. 3A-E). Importantly, differentiated cells displayed significantly lower ratios of acetylated tubulin-positive (*Ac-tub*^*+*^) cells (a marker of ciliated cells) compared with the control (21.29% vs. 37.64%, p < 0.001) (Fig. 3A-B, and Fig. 3F-H). The expression of NK2 homeobox 1 (*NKX2-1*), a specific marker of lung epithelial progenitors, was increased apparently in the stretched group compared with the control (39.51% vs. 14.06%, p < 0.0001) (Fig. 3I-K). However, there was no significant between-group difference in expression of differentiated secretory cell markers.

### Transcriptome profiling

#### Analysis of DEGs between stretched (S) and unstretched (N) groups

**Fig. 4:**
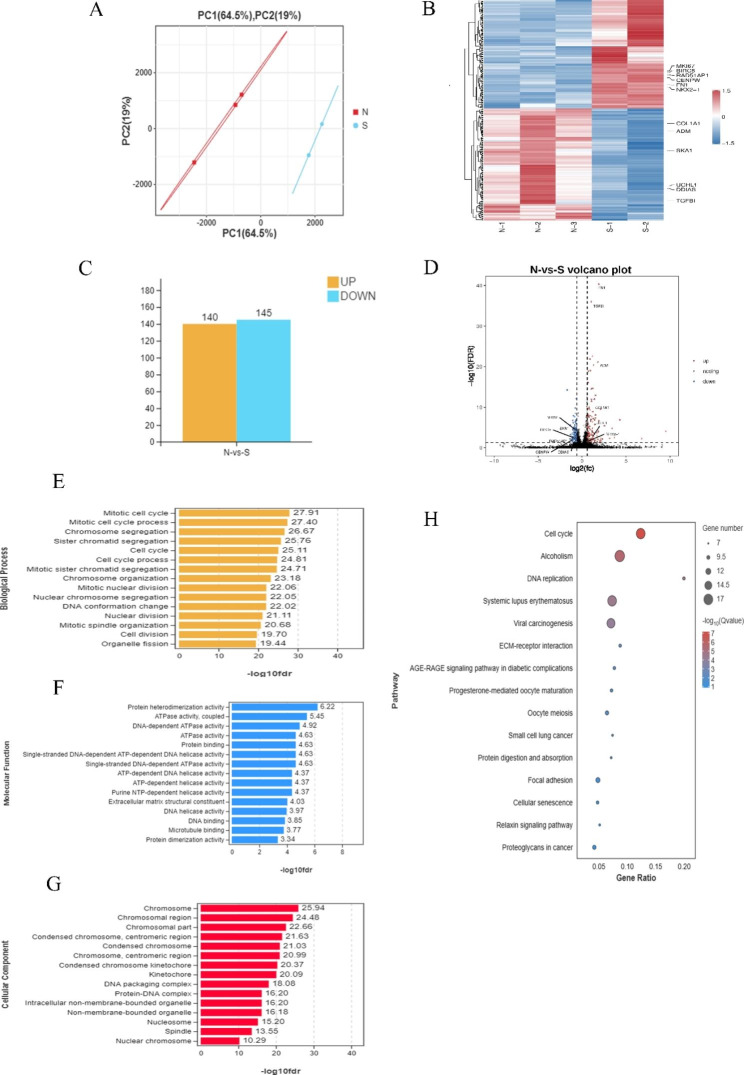
The overall transcriptomes characteristics between the control and stretch groups. (**a**) The PCA plot of transcriptome profiling. (**b**) Heat maps of mRNA (|log2FC| >0.58 and FDR < 0.05). Red and blue indicate up- and downregulation, respectively. (**c**) Histogram of differentially expressed genes (DEGs) expression between the control and stretch groups. (**d**) The volcano analysis of DEGs with color-coded. The x-axis represented the log2 of fold change (FC) and the y-axis represented log10 of p values. Blue dots were downregulated genes and red dots were upregulated genes. The leftover black dots were the genes without significant differences. The top 15 enrichment GO of differentially expressed genes. All GO terms were divided into three ontologies: yellow, biological process (**e**); blue, molecular function (**f**), and red, cellular component (**g**). The top 15 KEGG pathway terms (**h**) of differentially expressed genes

Although we have collected three biological replicates per group, one outlier sample in the stretched group was removed. Genes with a false discovery rate (FDR) < 0.05 and |logFC| > 0.58 were identified as DEGs. In total, 285 DEGs were screened between stretched (S) and unstretched (N) groups, among which 140 were upregulated and 145 were downregulated (Fig. 4A-D). The list of 285 DEGs is added as Additional file 2.

Gene Ontology (GO) was used to analyze significant DEGs between unstretched and stretched groups. Biological process results revealed that mechanical stretch mainly interfered with cell cycle processes, formation of chromosomes or DNA, and regulation of cell division. Molecular function results indicated enrichment of genes primally involved in binding and activity processes related to protein, ATP, and DNA; the constitution of extracellular matrix structure; and regulation of tubulin binding. Cellular component results indicate that the most affected genes were involved in chromosomes, nucleosomes, spindle, and DNA packing. The top 15 most significantly enriched GO terms are shown in Fig. 4E-G.

Kyoto Encyclopedia of Genes and Genomes (KEGG) pathway analysis revealed that DEGs are mainly enriched for the cell cycle (16 genes), DNA replication (8 genes), extracellular matrix-receptor interaction (8 genes), and focal adhesion (10 genes). In the pathway related to the cell cycle, *Ink4b*, which is related to the G1 phase, displayed increased expression. However, S and G2/M phase-related genes, such as *MCM, CDK1, CDC20*, showed decreased expression in the stretched group compared with the unstretched group. Our *vitro* results also showed that mechanical stretch could induce G1 arrest of ABSCs. The top 15 KEGG enrichment pathways are displayed in Fig. 4H. Expression of DEGs for the cell cycle pathway in KEGG analysis are provided in the Figure S2.

#### Validation of sequencing data by RT-qPCR

**Fig. 5:**
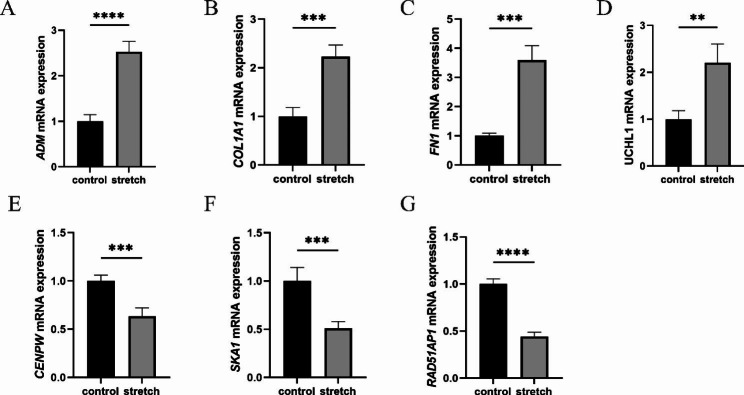
Quantitative RT-qPCR validation of differential genes. Statistical significance of expression level with *for p < 0.05, **for p < 0.01, ***for p < 0.001, and ****for p < 0.0001, compared with the control. Data are presented as the mean ± SD from at least three experiments

To verify the results of our sequencing data, we quantified the mRNA expression levels of eight genes using RT-qPCR, including ADM (the mechanical force-regulated gene), COL1A1 and FN1 (the main components of extracellular matrix), MKI67 (a marker of proliferation), RAD51AP1, SKA1, and CENPW (involved in mitotic cell cycle and DNA repair), and UCHL1 (the neural marker protein gene). The results are consistent with our transcriptome sequencing data. Specifically, we found that UCHL1, FN1, COL1A1, and ADM mRNA were upregulated (p = 0.0028, p = 0.0001, p = 0.0005, and p < 0.0001, respectively), while CENPW, RAD51AP1, SKA1, and MKI67 were downregulated (p = 0.0005, p < 0.0001, p = 0.0008, and p < 0.0001, respectively) in the stretched group compared with the unstretched group (Figs. 2C and 5).

#### Potential genes related to ABSCs’ function during mechanical stretch

**Fig. 6:**
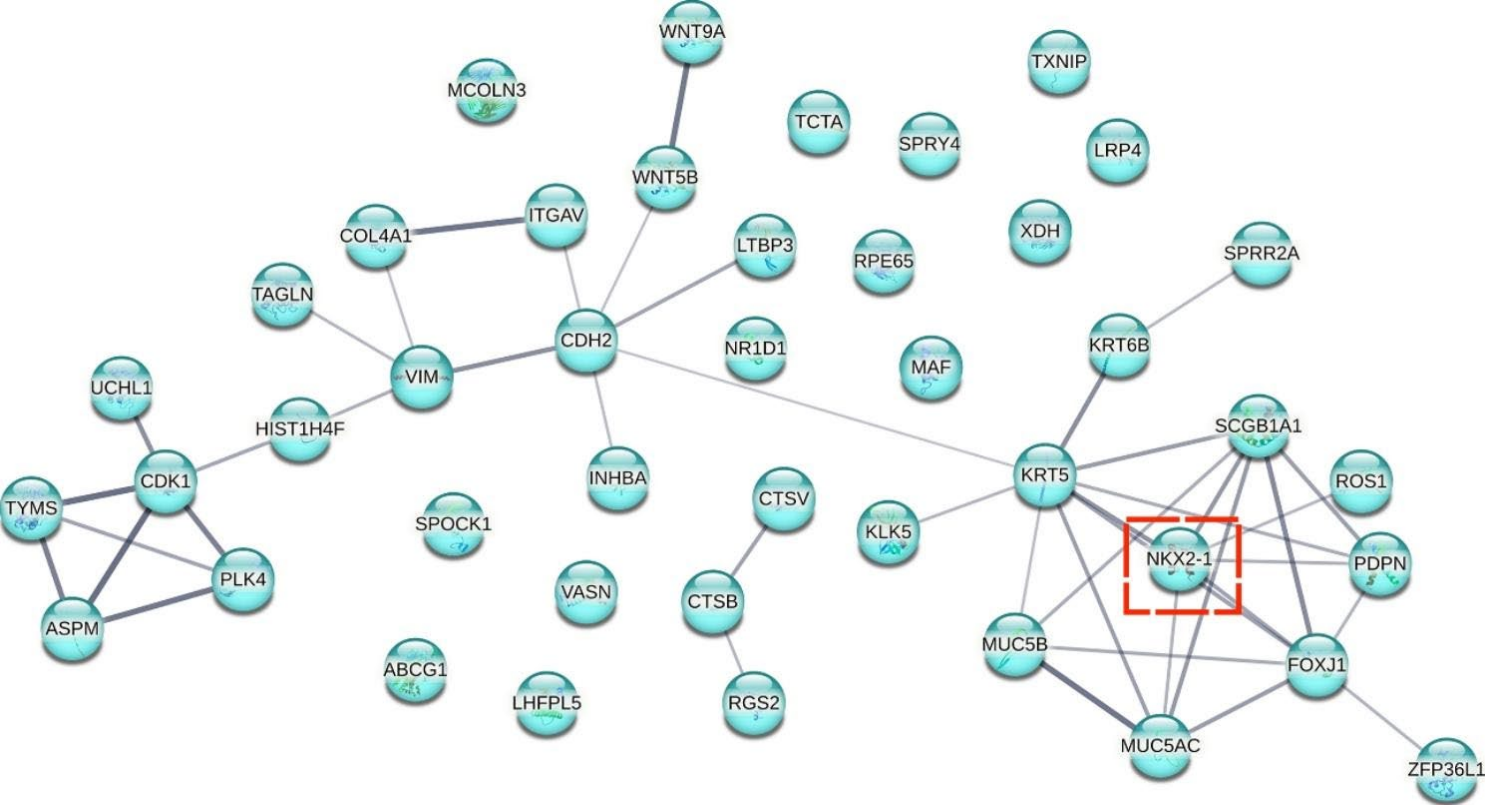
Analysis of protein-protein interaction networks of forty-two genes involved in epithelial cell differentiation. The line thickness indicates the strength of data support

**Fig. 7:**
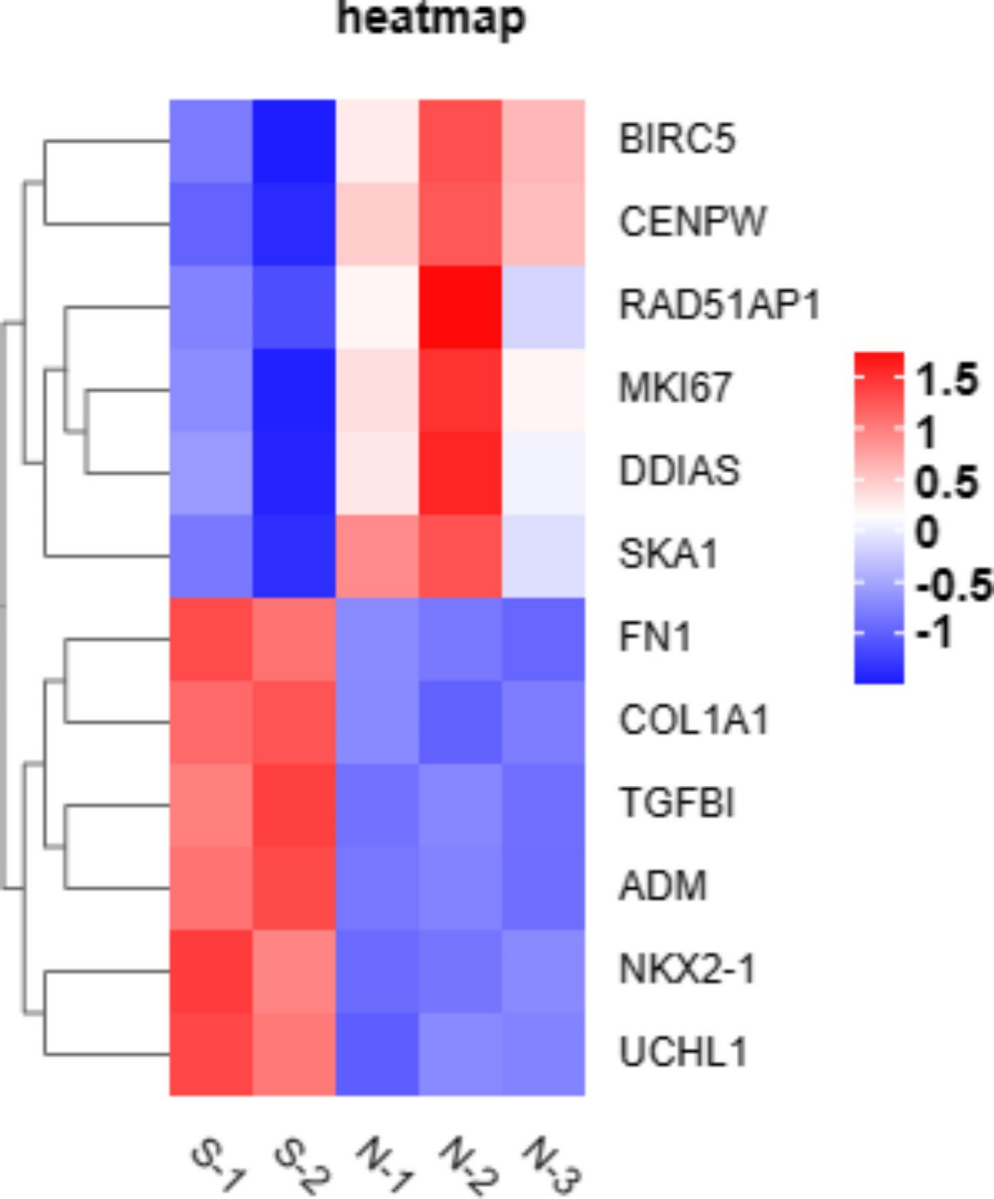
The heatmap for the potential genes related to the function of ABSCs during the mechanical stretch. Red and blue indicate up- and downregulation, respectively

Because we observed in vitro that mechanical stretch impaired the function of ABSCs by triggering apoptosis (Fig. 1), inhibiting proliferation (Fig. 2), and disrupting cilia cell differentiation (Fig. 3), we attempted to identify key genes that may play a potential role in ABSCs’ function among 285 DEGs. As for apoptosis, we noticed that DNA-damage-induced apoptosis suppressor (*DDIAS*) and baculoviral IAP repeat containing 5 (*BIRC5*) that have an anti-apoptotic function were both downregulated significantly in our transcriptome profiling, it suggested that *DDIAS* and *BIRC5* may involve the process of mechanical stretch-induced apoptosis in ABSCs. In addition, we observed that the transforming growth factor beta induced (*TGFBI*) which possibly played an crucial role in controlling the proliferation of ABSCs was upregulated remarkably when ABSCs were stretched. Finally, in our IF staining (Fig. 3I-K) and PPI analysis (Fig. 6), we revealed that NKX2-1 was up-regulated obviously and potentially involved in the differentiation of ABSCs during the mechanical stretch. The heatmap for these potential genes was provided as Fig. 7.

## Discussion

In this study, we found that prolonged mechanical stretching impaired ABSCs function, including induction of apoptosis, inhibition of proliferation, and dysfunction of cilia cell differentiation. Our transcriptome profiling supports our in vitro results and suggests that *DDIAS, BIRC5, TGFBI*, and *NKX2-1* might be the signaling molecules involved in the function of ABSCs during mechanical stretch. Mechanical stretch may be one of the reasons for abnormal repair of airway epithelium after injury. To our knowledge, ours is the first study focused on how mechanical stretch affects primary human ABSCs function, providing novel insight into the pathogenesis and treatment of chronic airway diseases with obviously disordered mechanical environments, such as asthma, bronchiectasis, and COPD.

During development of the whole airway lumen and lung, the airway continuously undergoes the dynamic mechanical forces of the lung. Airway epithelial cells have mechanically sensitive cell membrane channels capable of responding to changes in the mechanical environment and regulating airway behavior and structure [10, 26, 27]. Although growing evidence indicates that mechanical stress could affect airway epithelial cell function and phenotype, the biomechanical environment of ABSCs remains elusive. Using the Flexcell Tension System, Tschumperlin et al. discovered that mechanical strain with 17–22% elongation corresponded approximately to strains 37–50% of total lung capacity, which would damage cells [24]. On this basis, previous studies have referred to the mechanical parameters mentioned above to simulate the pathological mechanical environments in airways [16–18]. Therefore, we also used the Flexcell FX-5000 Tension System to establish a model of ABSCs mechanical injury with 20% elongation [24]. We found that prolonging the mechanical stretch of ABSCs activated apoptosis, hindered their proliferation, and impeded ciliogenesis. Apoptosis of ABSCs and caspase-3 activity were enhanced in time-dependent manners, especially following stretching for 48 h. Similar findings have been observed for other cell types. Juan et al. noticed that with prolonged states of mechanical stretch, even with 5% elongation, apoptosis was induced in fibroblasts [28]. Wu et al. also observed that mechanical stretch with 20% elongation could cause apoptosis of alveolar epithelial cells, and the severity of apoptosis increased over time [29]. The apoptosis of airway epithelial cells was observed in fetal sheep after prolonged mechanical ventilation [30]. Moreover, in our transcriptome profiling, we noticed that DDIAS and BIRC5 may involve the process of mechanical stretch-induced apoptosis in ABSCs. Previous studies have confirmed that high expression of DDIAS which has an anti-apoptotic function promotes cell survival [31, 32]. Besides, Brunette et al. found that DDIAS deficiency disturbed the process of homologous recombination, even breaking the DNA double-strand [33]. Similarly, our GO analysis revealed that mechanical stretch interfered with DNA packing, formation, or replication. Therefore, we proposed that mechanical stretch may induce the apoptosis of ABSCs through damaging DNA. An additional pro-apoptosis mechanism for ABSCs might be decreased expression of BIRC5, which was found decreased obviously in our RNA-seq. BIRC5 encodes for the protein survivin, blocking the final steps of the apoptotic pathway and inhibiting executioner caspases [34]. It has been proven that up-regulation of BIRC5 could impede the cell apoptosis-induced by cyclic stretch [35]. Maybe mechanical stretch induced the apoptosis of ABSCs by regulating the expression of DDIAS and BIRC5 which needs further exploration.

The disruption of airway epithelium homeostasis may lead to various pathological manifestations. Proliferation and differentiation are the most important functions of ABSCs (the vital progenitors of the airway) to maintain the dynamic balance of airway epithelium and repair after injury. Deptula et al. have found that mechanical ventilation for 15 min could cause airway epithelial injury which was repaired by activating ABSCs in the fetal, preterm lambs [36]. However, little is known about how mechanical force regulates ABSCs function. We first found that mechanical stretch for over 48 h hindered ABSCs proliferation and differentiation into ciliated cells. Many investigations have revealed that mechanical force had a strong influence on controlling the fate of airway epithelial cells [37, 38]. Saval et al. observed that mechanical force significantly slowed wound repair in human and cat airway epithelial cells [39]. Moreover, prolonged mechanical ventilation aggravated airway dysfunction in premature infants whose lungs were very immature [40]. Differing from the above studies, we used healthy primary human ABSCs for mechanical stimulation. Even in healthy ABSCs, overstretching impaired self-renewal. Our GO and KEGG analyses indicated enrichment of genes and pathways concentrated in regulation of cell cycle and cell division, further supporting our in vitro results. We also determined that TGFBI might be a signaling molecule and play a role in the proliferation of ABSCs during mechanical stretch. TGFBI protein is induced by TGF-β. Evidence showed that TGF-β plays an crucial role in controlling the fate of ABSCs. Inhibition of TGFβ/BMP/SMAD pathway signaling promoted ABSCs hyperplasia [41]. Kiyokawa et al. determined that TGF-β signaling could slow the cell cycle by suppressing Id2 in ABSCs [42]. Interestingly, elevated mechanical tension activated the TGF-β signaling pathway in alveolar stem cells, leading to pulmonary alveolar regeneration dysfunction [43]. Perhaps TGFBI was the potential gene involving ABSCs’ proliferation, more experiments are needed to verify this in the future. Collectively, these results suggest that long-term mechanical stretch probably slowed the proliferation of ABSCs by preventing their transition from G0/G1 to S phase.

The strength of mechanical force in regulating alveolar epithelial cell differentiation was demonstrated by Jiao Li et al. [11], who reported that during lung development, low-strength mechanical stimuli impeded differentiation of alveolar epithelial type I cells, while high-strength mechanical stimuli promoted differentiation of alveolar epithelial type I cells into alveolar epithelial type II cells. However, in the context of pulmonary fibrosis, persistent elevated mechanical force caused by alveolar regeneration dysfunction drove the progression of pulmonary fibrosis [12]. Runguang Li et al. demonstrated that mechanical stretch also inhibited mesenchymal stem cell differentiation into adipocytes [44]. Similarly, we observed that overstretched ABSCs were hard to differentiate into cilia cells. Airway cilia have the capacity to clear mucus and excrete inhaled contaminants. Dysfunctional or deficient cilia result in mucus plugs and severe lung diseases [45, 46]. Sanderson et al. confirmed that mechanical stimulation could affect ciliary beating activity, which was achieved by using a glass microprobe to stimulate the cell surface [47]. Therefore, disruption of ciliogenesis caused by overstretching might explain why mucus secretion increases in a sustained manner in patients with CAD, including individuals with COPD, asthma, and bronchiectasis. In addition, we noticed that the expression of *NKX2-1* was upregulated after stretching,which may be closely related to ABSCs differentiation. *NKX2-1* plays an essential role in normal airway system development. Disruption involving *NKX2-1* interrupted epithelial cell differentiation and morphogenesis, even causing the failure of lung development [48, 49]. We presumed that *NKX2-1* plays a potential role in the differentiation of ABSCs. However, the mechanism by which mechanical force affects ABSCs function needs further exploration.

In CAD, pathological mechanical stretching increased the expression of inflammatory mediators, including IL-8, IL-13, and MMP-9, and induced epithelial-mesenchymal transition in airway epithelial cells that exacerbated airway remodeling [15, 17, 18]. Based on our results verifying the sequencing data, we found that collagen type 1 (*COL1A1*) and fibronectin 1 (*FN1*), the main components of extracellular matrix, were highly expressed in the stretched group. We considered that mechanical stretch damage to ABSCs may contribute to extracellular matrix remodeling – the main pathological characteristic of CAD. Several studies determined that pathological mechanical stretch increased expression levels of collagen and fibronectin, thereby affecting airway remodeling [21, 50]. The same phenomenon was observed in a model of lipopolysaccharide-induced cell injury [51]. Our research also found the upregulation of several inflammatory genes (*CCL22, IL32*, and *MMP2*) in stretch groups, this suggested that mechanical stretch is probably involved in an inflammatory response of the airway. However, variations in cell type and mechanical loading parameters (such as extension, amplitude, frequency, and duration) may cause these stretch-induced inflammatory genes to deviate from previous studies [15, 17, 18]. Therefore, further validation of these results is required. Our results provide indirect evidence for the mechanism underlying abnormal repair of airway epithelium in individuals with CAD.

There are two primary limitations of our study. Once is that we did not observe the biomechanical effect of how pathological ABSCs respond to mechanical stretch, which possibly resembles the pathological status of patients with CAD. Another is that we did not study the impact of different mechanical loading parameters on ABSCs, which may provide optimal mechanical stimulation conditions for ABSCs growth and regeneration. Taken together, mechanical stretch damaged the function of healthy ABSCs and likely aggravated dysfunctional regeneration after airway injury.

## Conclusions

our results provide the first evidence that prolonged mechanical stretching time induces primary human ABSCs apoptosis, impedes ABSCs proliferation, and disrupts ciliary cell differentiation. RNA-Seq analysis supports our results and revealed that *DDIAS, BIRC5, TGFBI*, and *NKX2-1* may be closely related to the function of primary human ABSCs during mechanical stretch. Altogether, our findings suggest that mechanical stretching participates in abnormal regeneration and repair processes of airway epithelium in chronic airway diseases by disturbing ABSCs function.

### Electronic supplementary material

Below is the link to the electronic supplementary material.

**Additional file 1:** The supplementary materials of this study: The supplementary data of this study, including the identification of primary human ABSCs (Figure S1), the expression of DEGs in the cell cycle pathway with KEGG analysis (Figure S2 ), the annotated DEGs (CCL22, IL32, and MMP2) in the volcano plot (Figure S3), the primer sequences for RT-qPCR (Table S1), the information of patients (Table S2 ).

**Additional file 2**: The list of 285 differentially expressed genes (DEGs): A complete list of 285 DEGs, including gene ID, fold changes, p values, FDR values and description.

## Data Availability

The datasets used and analysed during the current study are available from the corresponding author on reasonable request.

## Abbreviations

CAD: chronic airway diseases
COPD: chronic obstructive pulmonary disease
ABSCs: Airway basal stem cells
RT-qPCR: quantitative real-time PCR
FDR: false discovery rate
DEGs: differentially expressed genes
KEGG: Kyoto Encyclopedia of Genes and Genomes
GO: Gene Ontology
*COL1A1*: collagen type 1
*FN1*: fibronectin 1
*NKX2-1*: NK2 homeobox 1
*ADM*: Adrenomedullin
*UCHL1*: ubiquitin C-terminal hydrolase L1
*CENPW*: centromere protein W
*RAD51AP1*: RAD51 associated protein 1
*SKA1*: spindle and kinetochore associated complex subunit 1
*TGBI*: transforming growth factor beta induced
*MKI67*: marker of proliferation Ki-67
*DDIAS*: DNA damage induced apoptosis suppressor
*BRIC5*: baculoviral IAP repeat containing 5
*CCL22*: C-C motif chemokine ligand 22
*IL32*: interleukin 32
*MMP2*: matrix metallopeptidase 2
