# Supplementary material for: Mechanical stretch promotes apoptosis and impedes ciliogenesis of primary human airway basal stem cells
Source: Respir Res. 2023 Sep 29;24:237. doi: 10.1186/s12931-023-02528-w (PMC10540374; doi:10.1186/s12931-023-02528-w)
Supplement: Supplementary file 1 — Additional file 1: The supplementary materials of this study: The supplementary data of this study, including the identification of primary human ABSCs (Figure S1), the expression of DEGs in the cell cycle pathway with KEGG analysis (Figure S2 ), the annotated DEGs (CCL22, IL32, and MMP2) in the volcano plot (Figure S3), the primer sequences for RT-qPCR (Table S1), the information of patients (Table S2 ). [file 12931_2023_2528_MOESM1_ESM.pdf]

Supplementary materials

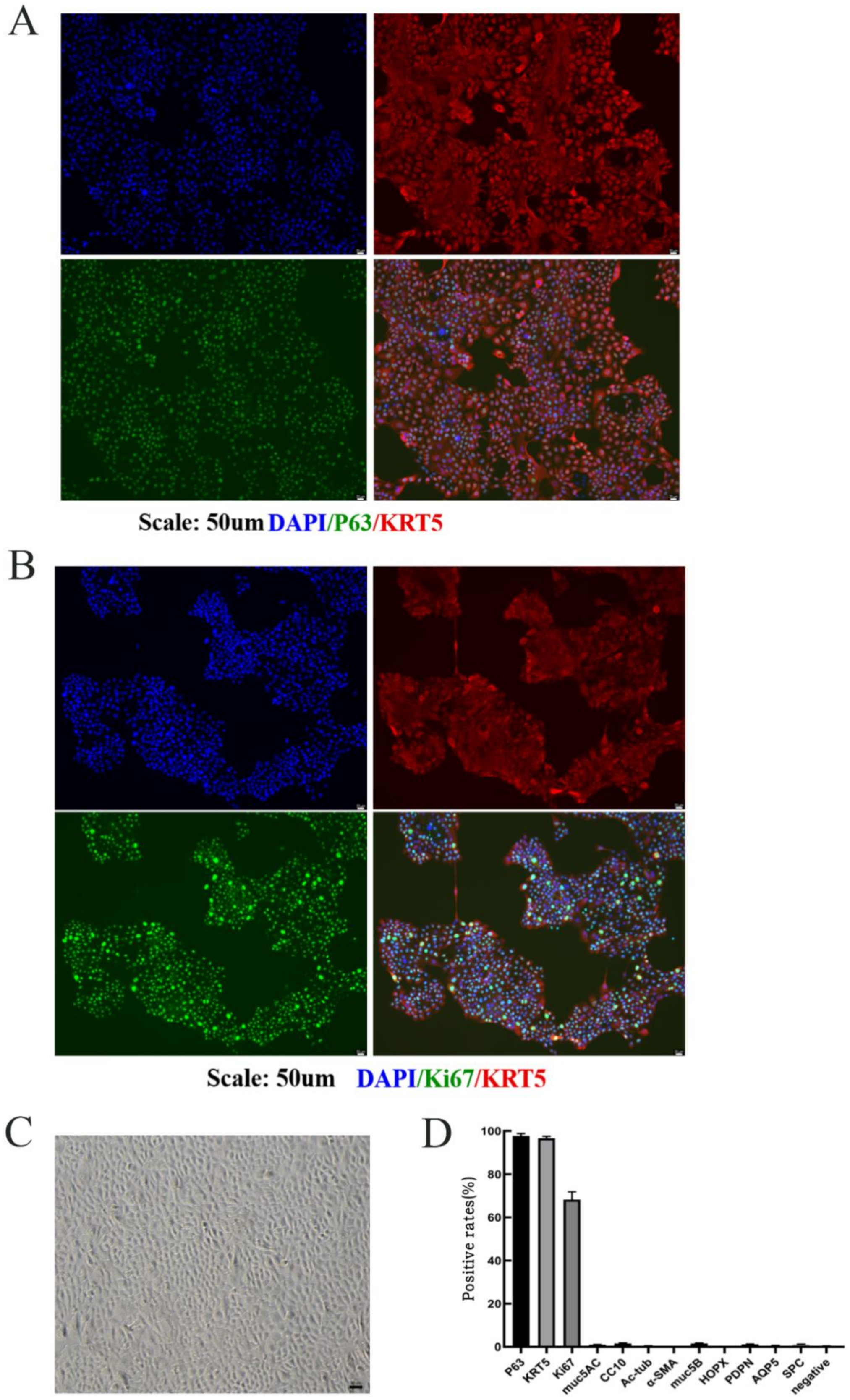

Figure S1 Identification of primary human airway basal stem cells (ABSCs). (A) ABSCs were stained with anti-tumor protein p63 (green), anti-keratin 5 (red), and DAPI (blue). (B) ABSCs were stained with anti-ki67(green), anti-keratin 5 (red), and DAPI (blue). (C) ABSCs presented structured and "slabstone"-like arrange under the microscope. (D) Over 90% of cells were positive for the markers of ABSCs in tumor protein p63 and anti-keratin 5. Over 60% of ABSCs were positive for the marker of proliferation ki67, and the expression of markers were negative in other types of epithelial cells. Scale bars represent 50um in (A), (B), and (C).

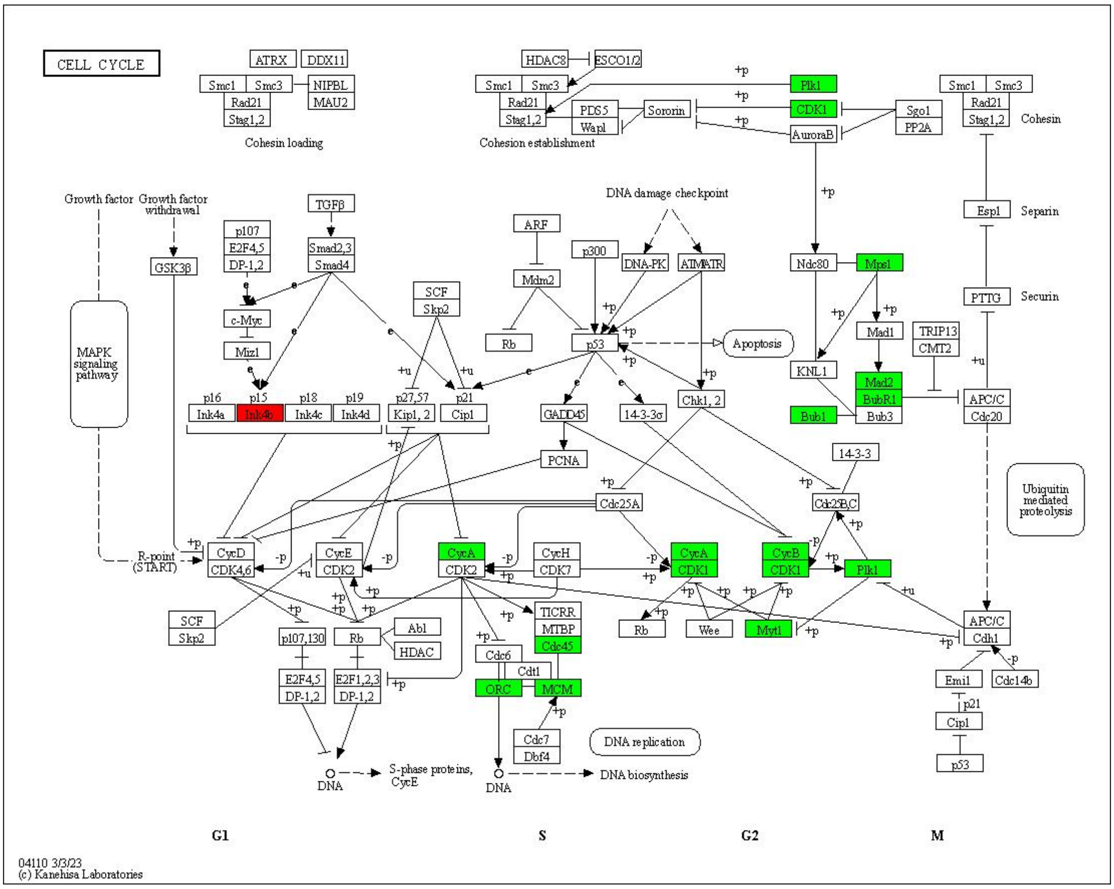

Figure S2 The expression of differentially expressed genes in the cell cycle pathway with KEGG analysis. The green box indicates the downregulated genes. The red box indicates the upregulated genes.

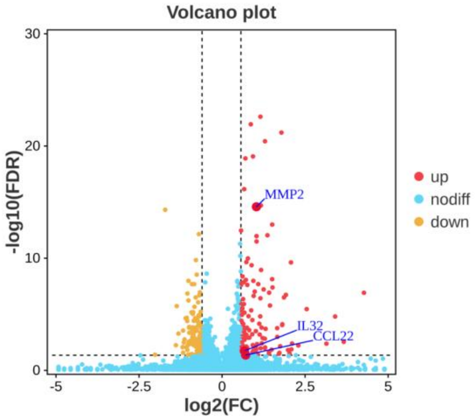

Figure S3 The volcano analysis of DEGs with color-coded. *CCL22, IL32*, and *MMP2* are annotated in the volcano plot.

Table S1 Primer sequences for RT- qPCR.

MKI67-F	CAAGAGGCAAATCATCCGAACCC
MKI67-R	TGTTCAGCTCTTCCGCAGGT
ADM-F	GGTTTCCGTCGCCCTGATGTA
ADM-R	AGCCCACTTATTCCACTTCTTTCG
CENPW-F	TACTGGCCGCAGCAAAGGTA
CENPW-R	GTTACCACCCAAAAGAATGCATCA
COL1A1-F	AAAGATGGACTCAACGGTCTC
COL1A1-R	CATCGTGAGCCTTCTCTTGAG
FN1-F	AATAGATGCAACGATCAGGACA
FN1-R	GCAGGTTTCCTCGATTATCCTT
RAD51AP1-F	TAATGCTTTGGTGACTTCGGT
RAD51AP1-R	AGGACTGCGTATTTCTAATGGT
SKA1-F	TTGACCCCAGTTCCTGACACA
SKA1-R	CTGAGTTATCCAGGTGCCCAT
UCHL1-F	GGCCAATGTCGGGTAGATGA
UCHL1-R	CATGGTTCACCGGAAAAGGC

Table S2 The information of patients.

Name	Sex	Age	Diagnosis	Comorbidities	Treatment	Pathological results
Patient 1	Male	48	Right lower pulmonary nodules	/	Minimally invasive surgery	Right lower lung granulomatous inflammation
Patient 2	Male	38	Right lower pulmonary nodules	/	Minimally invasive surgery	Right lower lung adenocarcinoma in situ (0.7cm)
Patient 3	Female	37	Right upper pulmonary nodules	/	Minimally invasive surgery	Right upper lung microinvasive adenocarcinomas (0.6cm)
Patient 4	Male	55	Right lower pulmonary nodules	/	Minimally invasive surgery	Right lower lung adenocarcinoma in situ (0.6cm)
